# Clinical parameters predicting pathologic complete response following neoadjuvant chemoradiotherapy for rectal cancer

**DOI:** 10.1186/s40880-015-0033-7

**Published:** 2015-08-13

**Authors:** Wei-Gen Zeng, Jian-Wei Liang, Zheng Wang, Xing-Mao Zhang, Jun-Jie Hu, Hui-Rong Hou, Hai-Tao Zhou, Zhi-Xiang Zhou

**Affiliations:** Department of Colorectal Surgery, Cancer Hospital, Chinese Academy of Medical Sciences, Peking Union Medical College, 17 Panjiayuan Nanli, Chaoyang District, Beijing, 100021 P.R. China; The Overall Planning Office, Cancer Hospital, Chinese Academy of Medical Sciences and Peking Union Medical College, Beijing, 100021 P.R. China

**Keywords:** Rectal cancer, Pathologic complete response, Neoadjuvant chemoradiotherapy, Carcinoembryonic antigen, Interval

## Abstract

**Introduction:**

Preoperative chemoradiotherapy (CRT), followed by total mesorectal excision, has become the standard of care for patients with clinical stages II and III rectal cancer. Patients with pathologic complete response (pCR) to preoperative CRT have been reported to have better outcomes than those without pCR. However, the factors that predict the response to neoadjuvant CRT have not been well defined. In this study, we aimed to investigate the impact of clinical parameters on the development of pCR after neoadjuvant chemoradiation for rectal cancer.

**Methods:**

A total of 323 consecutive patients from a single institution who had clinical stage II or III rectal cancer and underwent a long-course neoadjuvant CRT, followed by curative surgery, between 2005 and 2013 were included. Patients were divided into two groups according to their responses to neoadjuvant therapy: the pCR and non-pCR groups. The clinical parameters were analyzed by univariate and multivariate analyses, with pCR as the dependent variable.

**Results:**

Of the 323 patients, 75 (23.2%) achieved pCR. The two groups were comparable in terms of age, sex, body mass index, tumor stage, tumor location, tumor differentiation, radiation dose, and chemotherapy regimen. On multivariate analysis, a pretreatment carcinoembryonic antigen (CEA) level of ≤5 ng/mL [odds ratio (OR) = 2.170, 95% confidence interval (CI) = 1.195–3.939, *P* = 0.011] and an interval of >7 weeks between the completion of chemoradiation and surgical resection (OR = 2.588, 95% CI = 1.484–4.512, *P* = 0.001) were significantly associated with an increased rate of pCR.

**Conclusions:**

The pretreatment CEA level and neoadjuvant chemoradiotherapy-surgery interval were independent clinical predictors for achieving pCR. These results may help clinicians predict the prognosis of patients and develop adaptive treatment strategies.

## Background

Preoperative chemoradiotherapy (CRT), followed by total mesorectal excision (TME), has become the standard of care for patients with clinical stages II and III rectal cancer [1, 2]. Compared with surgical resection alone or postoperative CRT, preoperative CRT improves local control and increases the rate of sphincter preservation [3, 4]. However, the response to neoadjuvant CRT varies among different individuals. Most patients respond to neoadjuvant CRT, and approximately 10%–30% of patients achieve a pathologic complete response (pCR), wherein they lack any viable tumor cells in the final surgical specimen [5]. By contrast, some patients show no response or are resistant to CRT. It has been well documented that patients who achieved pCR had better long-term outcomes than those without pCR [5–8]. Moreover, a wait-and-see policy is safe and feasible for patients with clinical complete response to neoadjuvant CRT [9, 10].

As patients with pCR have a better prognosis, and the treatment strategy for these patients may be entirely different from that for the patients without pCR, the ability to predict the response to neoadjuvant CRT is of great clinical importance. Nevertheless, the factors that predict patient response to neoadjuvant CRT for rectal cancer have not been well defined. Several small retrospective studies have identified some clinical factors and molecular biomarkers that are predictors of tumor response to preoperative CRT, including the tumor size, carcinoembryonic antigen (CEA) level, epidermal growth factor receptor, and p21 [11–13]. These studies had small sample sizes of patients with pCR and investigated different response levels. Consequently, it remains a great challenge for clinicians to predict pCR after neoadjuvant CRT for rectal cancer.

In this study, we evaluated a large number of patients with pCR and aimed to indentify clinical factors or treatment variables associated with complete response to preoperative CRT for rectal cancer.

## Patients and methods

### Patient selection

This study was approved by the ethics committee at Cancer Hospital, Chinese Academy of Medical Sciences, and the protocol conformed to the ethical guidelines of the 1975 Declaration of Helsinki. A total of 348 consecutive patients who underwent neoadjuvant CRT between January 2005 and December 2013 were identified from our prospectively entered database. The criteria for neoadjuvant CRT were as follows: rectal adenocarcinoma confirmed by pathology, clinical stages II and III tumors, and tumors located within 10 cm of the anal verge. Twenty-five patients were excluded from the study for the following reasons: 2 patients refused surgery, 16 were treated with neoadjuvant radiotherapy without chemotherapy, and 7 underwent surgery at other hospitals.

### Evaluation

All patients underwent colonoscopy with biopsy and were histologically diagnosed with adenocarcinoma. Preoperative clinical staging was determined by abdominal and pelvic computed tomography (CT), transrectal ultrasonography, pelvic magnetic resonance imaging, or a combination of these.

At least two pathologists, who are specialized in colorectal cancer, assessed the surgical specimens. pCR was defined as the absence of viable tumor cells in the surgical specimen, including lymph nodes. Patients without pCR were grouped into the non-pCR cohort.

### Treatment

As previously reported [14, 15], all patients planned to undergo a total irradiation dose of 50.0 Gy to the pelvic area, delivered in 2.0-Gy fractions daily, five times per week for 5 weeks. One of the two chemotherapeutic regimens was delivered concurrently with radiotherapy as follows: oral capecitabine at a dose of 1,650 mg/m^2^ per day for 35 days, without weekend breaks, or oral capecitabine at a dose of 1,650 mg/m^2^ per day for 35 days plus intravenous oxaliplatin at a dose of 50 mg/m^2^ once weekly for 5 weeks. Surgical resection was planned for 6–8 weeks after the completion of preoperative CRT, irrespective of the response to CRT. TME was performed for each patient.

### Data collection

The following data were reviewed in our database: sex, age, body mass index (BMI), clinical TNM classification, tumor differentiation, tumor distance from the anal verge, radiation dose, pretreatment serum CEA level, chemotherapy regimen, and the interval between CRT and surgery.

### Statistical analysis

Nonparametric variables are presented as the median and range, and categorical variables are presented as the frequency with percentages. Continuous variables were analyzed with the Mann–Whitney *U* test, and categorical variables were analyzed with the Chi square test or Fisher’s exact test, when appropriate. Univariate and multivariate analyses were performed by using logistic regression and Cox proportional hazard ratios to identify factors that predict pCR. All statistical tests were two-sided, and a *P* value of <0.05 was considered statistically significant. Data were analyzed by Statistical Package for the Social Science (SPSS) 18.0 for Windows (SPSS Inc., Chicago, IL, USA).

## Results

### Patient demographics

Patient demographics and tumor characteristics are shown in Table 1. A total of 323 consecutive patients with clinical stages II and III rectal adenocarcinoma who underwent long-course neoadjuvant CRT followed by TME were included in this study. The median age was 57 years (range 26–86 years). Of the 323 patients, 174 (53.9%) were males, and 149 (46.1%) were females; 78 (27.2%) had clinical stage II disease, and 235 (72.8%) had clinical stage III disease. The median distance of the tumor from the anal verge was 6 cm (range 0–10 cm). All patients received a long-course radiation, and the median radiation dose was 50 Gy (range 38–60 Gy). A total of 267 (82.7%) patients received oral capecitabine alone, concurrent with radiotherapy, and 56 (17.3%) patients received both capecitabine and oxaliplatin. The median interval between the completion of CRT and surgery was 50 days (range 25–105 days).

**Table 1:** Patient demographics and tumor characteristics

Variable	Total (*n* = 323)	pCR (*n* = 75)	Non-pCR (*n* = 248)	*P* value
Age (years)^a^	57 (26–86)	57 (26–86)	58 (32–78)	0.773
Sex				0.342
Male	174 (53.9)	44 (58.7)	130 (52.4)	
Female	149 (46.1)	31 (41.3)	118 (47.6)	
BMI (kg/m^2^)^a^	23.6 (17.0–32.2)	24.2 (17.4–31.2)	23.5 (17.0–32.2)	0.386
cT category				0.297
2	26 (8.0)	7 (9.3)	19 (7.6)	
3	234 (72.5)	58 (77.3)	176 (71.0)	
4	63 (19.5)	10 (13.3)	53 (21.4)	
cN category				0.791
N0	88 (27.2)	21 (28.0)	67 (27.0)	
N1	161 (49.9)	35 (46.7)	126 (50.8)	
N2	74 (22.9)	19 (25.3)	55 (22.2)	
cTNM classification				0.867
II	88 (27.2)	21 (28.0)	67 (27.0)	
III	235 (72.8)	54 (72.0)	181 (73.0)	
Distance from the anal verge (cm)^a^	6 (0–10)	6 (0–10)	6 (0–10)	0.721
Pretreatment CEA (ng/mL)^a^	3.8 (0.02–31.5)	2.9 (0.06–24.9)	4.2 (0.02–31.5)	0.001
Tumor differentiation				0.679
Well	24 (7.4)	6 (8.0)	18 (7.3)	
Moderate	250 (77.4)	60 (80.0)	190 (76.6)	
Poor	49 (15.2)	9 (12.0)	40 (16.1)	
Type of surgery				0.543
Low anterior resection	190 (58.8)	40 (53.3)	150 (60.5)	
Abdominoperineal resection	122 (37.8)	32 (42.7)	90 (36.3)	
Hartmann operation	11 (3.4)	3 (4.0)	8 (3.2)	
Chemotherapy				0.729
Capecitabine	267 (82.7)	61 (81.3)	206 (83.1)	
Capecitabine + oxaliplatin	56 (17.3)	14 (18.7)	42 (16.9)	
Time interval (day)^a^	50 (25–105)	57 (35–79)	49 (25–105)	<0.001
Radiation dose (Gy)^a^	50 (38–60)	50 (40–60)	50 (38–60)	0.225

*BMI* body mass index, *CEA* carcinoembryonic antigen, *pCR* pathologic complete response, *cT* *category* clinical tumor category, *cN category* clinical node category, *cTNM* *classification* clinical tumor-node-metastasis classification.

^a^These values are presented as the median followed by the range in parentheses; other values are presents as numbers of patients followed by the percentage in parentheses.

Of the 323 patients, 75 (23.2%) achieved a pCR, and 248 (76.8%) did not. The patients were divided into two groups, the pCR (*n* = 75) and non-pCR (*n* = 248) groups. The following clinical parameters were comparable between the pCR and non-pCR groups: age, sex, BMI, distance of the tumor from the anal verge, pretreatment clinical T or N category, and tumor differentiation. The chemotherapy regimens, radiation dose, and type of surgery were not significantly different between the pCR and non-pCR groups. The pretreatment serum CEA level was significantly lower in the pCR group than in the non-pCR group (2.9 vs. 4.2 ng/mL, *P* = 0.001). The median interval between the completion of CRT and surgery was significantly longer in the pCR group than in the non-pCR group (57 vs. 49 days, *P* < 0.001).

### Predictive factors of pCR

For the univariate and multivariate analyses, the variables were analyzed as discrete categorical variables. A median age of 57 was chosen as the cut-off value. We chose to investigate tumors located within 5 cm of the anal verge because that criterion could distinguish between middle and lower rectal cancer. An interval from the completion of CRT to surgery of longer than 7 weeks was chosen because it was the median interval in our study.

On univariate analysis, an interval from the completion of chemoradiation to surgery of >7 weeks was associated with an increased rate of pCR [odds ratio (OR) = 2.658, 95% confidence interval (CI) = 1.484–4.512, *P* = 0.001]. A lower pretreatment serum CEA level (≤5 ng/mL) was also significantly associated with an increased rate of pCR (OR = 2.249, 95% CI = 1.251–4.046, *P* = 0.007). Other factors, such as age, sex, tumor differentiation, clinical TNM classification, tumor distance from the anal verge, radiation dose, and chemotherapy regimen, were not significantly associated with pCR (Table 2).

**Table 2:** Univariate analyses of predictors for pCR

Variable	pCR (*n* = 75)	Non-pCR (*n* = 248)	Odds ratio (95% CI)	*P* value
Age (years)				
≤57	36 (48.0)	127 (51.2)	Reference	–
>57	39 (52.0)	121 (48.8)	1.137 (0.678–1.907)	0.626
Sex				
Male	44 (58.7)	130 (52.4)	Reference	–
Female	31 (41.3)	118 (47.6)	0.776 (0.460–1.309)	0.342
cT category				
2	7 (9.3)	19 (7.7)	Reference	–
3	58 (77.3)	176 (71.0)	0.894 (0.358–2.236)	0.811
4	10 (13.3)	53 (21.4)	0.512 (0.171–1.537)	0.233
cN category				
N0	21 (28.0)	67 (27.0)	Reference	–
N1	35 (46.7)	126 (50.8)	0.886 (0.478–1.642)	0.701
N2	19 (25.3)	55 (22.2)	1.102 (0.539–2.255)	0.790
cTNM classification				
II	21 (28.0)	67 (27.0)	Reference	–
III	54 (72.0)	181 (73.0)	0.952 (0.535–1.695)	0.867
Distance from the anal verge (cm)				
>5	44 (58.7)	156 (62.9)	Reference	–
≤5	31 (41.3)	92 (37.1)	1.195 (0.705–2.023)	0.508
Pretreatment CEA (ng/mL)				
>5	18 (24.0)	103 (41.5)	Reference	–
≤5	57 (76.0)	145 (58.5)	2.249 (1.251–4.046)	0.007
Tumor differentiation				
Well	6 (8.0)	18 (7.3)	Reference	–
Moderate	60 (80.0)	190 (76.6)	0.947 (0.360–2.495)	0.913
Poor	9 (12.0)	40 (16.1)	0.675 (0.209–2.182)	0.511
Type of surgery				
Low anterior resection	40 (53.3)	150 (60.5)	Reference	–
Abdominoperineal resection	32 (42.7)	90 (36.3)	1.333 (0.782–2.273)	0.290
Hartmann operation	3 (4.0)	8 (3.2)	1.406 (0.357–5.545)	0.626
Chemotherapy				
Capecitabine	61 (81.3)	206 (83.1)	Reference	–
Capecitabine + oxaliplatin	14 (18.7)	42 (16.9)	1.126 (0.577–2.198)	0.729
Time interval (weeks)				
≤7	23 (30.7)	134 (54.0)	Reference	–
>7	52 (69.3)	114 (46.0)	2.658 (1.532–4.609)	0.001
Radiation dose (Gy)				
<50	4 (5.3)	14 (5.6)	Reference	–
=50	63 (84.0)	221(89.1)	0.998 (0.317–3.138)	0.997
>50	8 (10.7)	13 (5.2)	2.154 (0.522–8.892)	0.289

All values are presents as numbers of patients followed by percentages in parentheses. Other footnotes as in Table 1.

*CI* confidence interval.

On multivariate analysis, a pretreatment CEA level of ≤5 ng/mL (OR = 2.170, 95% CI = 1.195–3.939, *P* = 0.011) and an interval from the completion of neoadjuvant CRT to surgery of >7 weeks (OR = 2.588, 95% CI = 1.484–4.512, *P* = 0.001) were identified as independent predictors for achieving a pCR (Table 3).

**Table 3:** Multivariate analyses of predictors for pCR

Variable	Odds ratio	95% CI	*P* value
Serum CEA (ng/mL)			
>5	Reference	–	–
≤5	2.170	1.195–3.939	0.011
Time interval (weeks)			
≤7	Reference	–	–
>7	2.588	1.484–4.512	0.001

Footnotes as in Tables 1 and 2.

## Discussion and conclusions

In this study, we analyzed clinical factors or treatment variables that are associated with a complete response to preoperative CRT. Our univariate and multivariate analysis results showed that a pretreatment CEA level of ≤5 ng/mL and an interval from the completion of neoadjuvant CRT to surgery of >7 weeks were significantly associated with an increased rate of pCR.

Few studies have evaluated clinical factors associated with complete response to preoperative CRT for rectal cancer [16–20]. García-Aguilar et al. [16] studied the clinical factors of 168 patients with locally advanced rectal cancer, but the authors failed to find any factors that were associated with pCR. Kalady et al. [17] identified that an interval of >8 weeks between the completion of preoperative CRT and surgical resection was the only predictor for pCR in a retrospective review of 242 patients. In the largest sample size study to date, Das et al. [18] evaluated the predictors of pCR in 562 patients and found that the circumferential tumor extent was the only factor that was significantly associated with pCR. In a group of 249 patients assessed by Park et al. [19], the pre-CRT movability, post-CRT size, post-CRT morphology, and gross change were independent predictors of pCR. More recently, Garland et al. [20] found that the tumor size and pretreatment clinical N category were independent predictors of pCR after evaluating the clinical factors of 297 patients.

The serum CEA level is widely used as a tumor marker in patients with colorectal cancer. The pretreatment CEA level is useful for assessing the prognosis, and postoperative CEA testing is used for the early detection of recurrent disease [21]. However, few studies have evaluated the value of the CEA level in predicting the response to preoperative CRT. We found that the CEA level was significantly higher in the non-pCR group than in the pCR group; 76.0% of the patients with a pCR had a normal pretreatment CEA level, versus 58.5% of the patients in the non-pCR group. In addition, a normal pretreatment CEA level was significantly associated with pCR in both univariate and multivariate analyses. Several previous studies have reported similar results [20, 22, 23]. Yoon et al. [22] found that the pretreatment CEA level was an independent predictor for pCR. Recently, Garland et al. [20] identified that the pretreatment serum CEA levels and a decrease in the pre- to post-treatment serum CEA level were significantly associated with pCR in univariate analysis. Additionally, tumor cells that have a high density of CEA may resist radiation [24]. However, the exact mechanism is unclear and remains to be elucidated.

Radiation-induced necrosis and subsequent tumor regression is a time-dependent phenomenon in which a longer interval between the completion of CRT and surgery may increase the rate of pCR [17]. In this study, we found that an interval of >7 weeks between the completion of CRT and surgical resection was significantly associated with a higher rate of pCR (31.3 vs. 14.6%, *P* = 0.001). Similarly, Kalady et al. [17] reported that an interval of >8 weeks was the only independent predictor for pCR (OR = 2.63, 95% CI = 1.13–6.12, *P* = 0.020). Wolthuis et al. [25] reported that an interval of >7 weeks was associated with increased pCR (28 vs. 16%, *P* = 0.030), and the 5-year cancer-specific survival rate was higher in the long-interval group than in the short-interval group (91 vs. 83%, *P* = 0.046). Conversely, Stein et al. [26] and Lim et al. [27] did not find that a longer interval between CRT and surgical resection was an independent predictor of pCR. To settle these disputes about the optimal time from CRT to surgery, randomized controlled trials are needed.

Increasing data indicate that the treatment response to chemoradiation associated with the oncologic outcomes [28]. To improve the response, some randomized trials have added oxaliplatin or targeted drugs into the currently widely used fluorinated, pyrimidine-based preoperative chemotherapy regimen [29–31]. However, none of these studies reported an increased pCR rate. Recently, O’Connell et al. [29] reported that adding oxaliplatin did not improve the rates of pCR, sphincter-sparing surgery, or surgical down-staging; instead, oxaliplatin added significant toxicity. We also found that the addition of oxaliplatin did not increase the pCR rate (25.0 vs. 22.8%, *P* = 0.729). By contrast, waiting a longer time between CRT and surgery was significantly associated with a higher rate of pCR.

There are some potential limitations of this study. Because this was a retrospective study, there may be bias. Additionally, we evaluated patients who were treated over the course of 9 years. The CRT regimen and pathologic assessment may change over time. Other studies have reported some variables that were not included in this study, such as tumor size, circumferential extent of tumor, morphology, and gross change after CRT. These factors are subjective parameters that are susceptible to inter-observer variations, and it is difficult to assess these factors in a retrospective study.

Further prospective studies are needed to identify the predictors for pCR and elucidate its potential mechanisms. In the future, it may be possible to stratify patients into different response groups before CRT is initiated, which could allow us to develop individualized therapy plans. Patients who are considered good responders to preoperative CRT may benefit little from TME, and their optimal treatment may involve nonoperative management or local excision. Patients who are stratified into the poor response group may need more aggressive therapies or alternative new therapies.

In conclusion, this large, retrospective study demonstrated that a pretreatment CEA level of ≤5 ng/mL and an interval from the completion of neoadjuvant CRT to surgery of >7 weeks were independent clinical predictors for achieving pCR. These findings may help clinicians predict the prognosis of patients and develop individualized treatment strategies.
